# Reply to “Quality control requirements for the correct annotation of lipidomics data”

**DOI:** 10.1038/s41467-021-24985-x

**Published:** 2021-08-06

**Authors:** Catherine G. Vasilopoulou, Karolina Sulek, Andreas-David Brunner, Ningombam Sanjib Meitei, Ulrike Schweiger-Hufnagel, Sven W. Meyer, Aiko Barsch, Matthias Mann, Florian Meier

**Affiliations:** 1 Max Planck Institute of Biochemistry, Martinsried, Germany; 2 NNF Center for Protein Research, Copenhagen, Denmark; 3 Ningombam Angouton Memorial Trust, Imphal East, Manipur India; 4 Luhup Private Limited, Indore, Madhya Pradesh India; 5 Bruker Daltonics GmbH & Co. KG, Bremen, Germany

**Keywords:** Lipidomics, Mass spectrometry, Data processing

**Replying** **to** H. Köfeler et al. *Nature Communications* 10.1038/s41467-021-24984-y (2021)

In Vasilopoulou et al.^1^, we reported the acquisition of over 100 fragment mass spectra per second at very high sensitivity in liquid chromatography-mass spectrometry (LC-MS)-based analyses of small biomolecules with trapped ion mobility spectrometry (TIMS) and parallel accumulation—serial fragmentation (PASEF). Our findings demonstrated advantages for lipidomics research as the method fully characterizes the vast majority of all detectable features in multiple dimensions (retention time, mass-to-charge, ion mobility, ion abundance, and fragment spectra), even in single LC-MS experiments. As a first step in interpreting this particularly dense data, we matched each fragment spectrum to an in silico database containing structure-specific ions and evaluated spectrum matches as fully detailed in the original publication. Köfeler et al. comment that additional criteria such as chromatographic behavior could be useful to manually refine the annotation of lipid structures, spectrum by spectrum. To set a precedent for their more general points, the commenters highlight common challenges in the annotation of lipids, applying criteria that go beyond the scope of our original study^2,3^. Here, we clarify important aspects of our work and argue for more innovative software solutions to make lipidomics accessible to a broader community.

The analysis of lipidomics data is a challenging task and diverse tools and workflows are used by the community, ranging from manual examination to software-based approaches^2^. In a laudable attempt to harmonize workflows, the members of the Lipidomics Standards Initiative, many of whom are authors of this Matters Arising article, set out to develop guidelines^3^. We share these goals and have promoted similar guidelines for many years in the proteomics community. However, at the time of the publication of the original article, this was and still is an ongoing process, not peer-reviewed, and actively discussed in the community (https://lipidomicssociety.org/interest_groups/lipidomics-standards-initiative-lsi/, accessed on October 16, 2020). To this end, we had transparently reported our data analysis steps and the criteria underlying our manual inspection of spectrum matches. Further, we had released all raw data and provided extensive Supplementary Data which include all layers of gradual evidence for each lipid annotation, making sure that researchers interested in particular lipids can ascertain the confidence level in each case or re-process the data. It is expected in an untargeted approach, and an inevitable part of omics disciplines, that such lists contain a fraction of potentially false-positive annotations, which turns the bioinformatic challenge into developing methods to reliably estimate the proportion of potentially false assignment, as has long been the case in proteomics. In contrast, it is not clear how the approach advocated by Köfeler et al.—up to the detailed manual inspection of all spectra by experts and even the synthesis of reference molecules—should be the future of omics-type investigations. In any case, facilitating data access allowed others to independently validate our collisional cross section (CCS) measurements^4^, which puts the commenters’ main concern into question.

Köfeler et al. elaborate on chromatographic characteristics of lipids and, in particular, the equivalent carbon number (ECN) model^5^. The Supplementary Data files of our original study list detected features with their experimental evidence and, based on this, we proposed an annotation for the associated fragment ion (MS/MS) mass spectra. The analysis by Köfeler et al. does not take into account our clearly stated choice to not collapse or remove lipid annotations if they were detected at multiple retention times sharing the same fragment ion characteristics, because they potentially could be isomers.

To address this point of criticism in more detail, we inspected the nature of these features. Taking the very first panel as an example, we reproduced Fig. 1 from Köfeler et al., but now additionally visualizing the relative intensity as well as the collisional cross section (Fig. 1a). This analysis revealed that the most abundant features indeed follow a nearly linear trend in accordance with the ECN retention time model. Interestingly, while some low-abundance features have deviating retention times, their collisional cross sections are virtually identical and the main fragment ions support our original annotation (Fig. 1b). Observing multiple chromatographic features with the same MS/MS-based lipid annotation was recently highlighted by some of the Matters Arising authors as a subject of future research^6^. Note that this effect could be even more pronounced with nanoflow chromatography (as used in our original study) due to its very high sensitivity. Some of these features might be biologically relevant and others technical artefacts, but we think it is valuable to acquire such data in the first place and hence we kept these annotations in the context of our technology-focused study. This does not imply that all of these features are true isomers, in particular if their number exceeds the number of biologically expected or possible isomers as in the example in Fig. 1 and as Köfeler et al. pointed out for 21 annotated spectra. In contrast to the commenters’ take, we think this highlights the power of PASEF to acquire informative MS/MS spectra, even for the least abundant features.

**Fig. 1:**
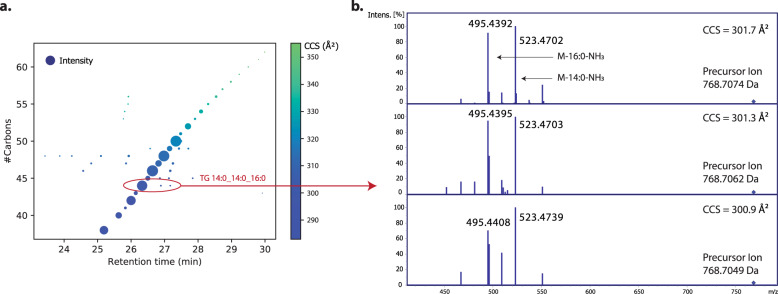
Retention time analysis of triacylglycerols (TGs) with 0 double bonds. **a** Retention time versus the number of fatty acyl carbons. Dot size indicates the relative ion intensity and dot color visualizes the collisional cross section (CCS). **b** Experimental MS/MS spectra of the three features highlighted in panel a supporting the annotation of TG14:0_14:0_16:0.

We certainly agree that the retention time is valuable information and can hint at potentially false-positive hits such as in-source fragments commonly observed in lipidomics. The putative late-eluting diacylglycerol is such an example. In practice, several strategies have been proposed to consider retention time information in the annotation step^5,7–10^, yet there is no consensus in the literature on which model should be used and examples of lipid annotations that do not strictly follow the ECN model are frequently encountered, even in studies published by some of the Matters Arising authors (Supplementary Fig. 1). Here, we chose two readily applicable strategies based on lipid subclass-specific elution windows that scale to large datasets. Depending on the parameters and which model is chosen, we found that 90–95% of our lipid annotations fall into “allowed” retention time ranges (Supplementary Fig. 2).

Köfeler et al. raise concerns regarding certain lipid subclasses, which are partially redundant and apply to only a minor fraction of our plasma dataset. Due to the brevity of this format, we address their more specific points in Supplementary Note 1. We note that some are simple misunderstandings. For example, we did not annotate the sphingomyelin “SM d16:1_25:0”, but used the recommended shorthand notation SM d41:1 in the manually annotated ‘lipid name’ column. The annotation “SM d16:1_25:0” is only shown in the “LSI ID” column, which contains the raw software output and was the underlying reason for this misunderstanding. Similarly, the mass overlap of phosphatidylcholine (PC) and SM isotopes is not a plausible source of error because we annotated the lipid spectra after four-dimensional feature detection and monoisotopic mass determination, and with a precursor mass tolerance of 5 ppm. Quite on the contrary, we think PCs and SMs showcase the added value of the ion mobility dimension, as they cluster separately in this space (Fig. 2) and with PASEF, distinct MS/MS spectra are acquired for mobility-resolved precursors.

**Fig. 2:**
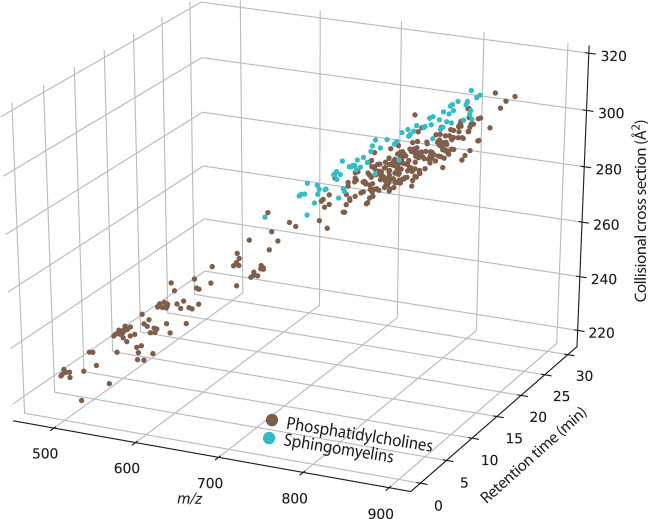
Lipid detection in nanoflow LC-TIMS-MS experiments. Separation of features in a human plasma extract annotated as phosphatidylcholine (PC) and sphingomyelin (SM) lipids in *m/z*, retention time, and ion mobility dimensions. One outlier is not shown because its CCS value is out of bounds.

With regards to ion mobility, it is interesting to compare CCS values of different molecular adducts. We thank Köfeler et al. for pointing out chemically implausible annotations of two diacyl phosphatidylinositols and 30 acetate adducts. However, we disagree that molecular adducts other than the expected dominant form should be disregarded per se and note that they are frequently reported in the literature^2^.

The Matters Arising article further refers to details of spectral annotations. Akin to metabolomics, informatics approaches in lipidomics typically employ (in silico) spectral libraries^11^. The manual inspection by Köfeler et al. highlighted current limitations of this approach, including the overly detailed annotation of sterols (which we only referred to as “cholesterol and derivates” for this reason) or the annotation of two chemically implausible fragments contained in the library. Rule-based decision-tree annotations are a promising alternative^12^, and most recently developed software even combines both approaches^13,14^. However, rule sets are also not unequivocal, often instrument-dependent, and typically compiled from different sources. While we are now actively working in this direction, such tools were not available for PASEF data at the time and we aimed to contribute to their developments by making data easily accessible. In the original article, we manually inspected the software-based spectrum matches as described in the Methods section and discussed with the reviewers to their satisfaction (see Peer Review file in ref. ^1^). To clarify, in positive mode, we observed the head group fragment (*m/z* 184.07) for 291/296 PCs and kept five additional annotations for which we observed the corresponding neutral loss of phosphocholine. Likewise, and contradicting Köfeler et al., we confirm that phosphatidylethanolamine (PE) and phosphatidylinositol (PI) species were identified based on their neutral losses (column P in Supplementary Data 2 of our original article^1^). In negative mode, we based our annotations on verifying fatty acyl fragments and accurate mass as explicitly stated in the Methods section. The comment by Köfeler et al. regarding characteristic fragment ions is thus not applicable.

As we had hoped, bioinformaticians are rapidly picking up on PASEF data and new tools are emerging for metabolomics and lipidomics^13,15^. This includes MS-DIAL, which, amongst other things, scores CCS values and integrates decision-tree algorithms to increase the confidence in lipid annotations^13^. We now re-processed our plasma raw data with the default parameters and collapsed the results to unique annotations on the species level (Supplementary Data 1). Reassuringly, this yielded 550 hits from positive and negative mode that passed the annotation criteria for CCS, precursor *m/z*, and MS/MS spectrum, with a similar lipid class distribution as in our initial report of 456 species-unique annotations (Supplementary Fig. 3). Note that these numbers are dwarfed by the close to 200,000 MS/MS spectra that can be acquired with PASEF in short gradients. Resolving the identity of all these unannotated features will be an exciting task for future research, which can only be tackled by combining the highest data quality with innovative bioinformatic approaches. Therefore, and based on the diversity of approaches to analyze lipidomics data evident from the work of ourselves, the commenters, and others, we could not agree more that data analysis remains a major bottleneck in lipidomics and that the community still has to establish widely accepted standards.

## Reporting summary

Further information on research design is available in the Nature Research Reporting Summary linked to this article.

## Supplementary information

Supplementary Information

Description of Additional Supplementary Files

Supplementary Data 1

Reporting summary

## Data Availability

The mass spectrometry raw data associated with the original article^1^ are accessible via the Mass Spectrometry Interactive Virtual Environment (MassIVE) with the dataset identifier MSV000083858 [10.25345/C51063], and processed data are available as Supplementary Data with the original article. The result file from the MS-DIAL analysis underlying Supplementary Fig. 3 is provided in Supplementary Data 1.
