# Supplementary material for: Reply to “Quality control requirements for the correct annotation of lipidomics data”
Source: Nat Commun. 2021 Aug 6;12:4772. doi: 10.1038/s41467-021-24985-x (PMC8346504; doi:10.1038/s41467-021-24985-x)
Supplement: Supplementary file 1 — Supplementary Information [file 41467_2021_24985_MOESM1_ESM.pdf]

Supplementary Information

Vasilopoulou et al., Reply to: ‘Processing of lipidomic data sets needs quality control’

Table of Contents

- Supplementary Figures 1-3.
- Supplementary Note 1.
- Supplementary References.

Supplementary Figure 1

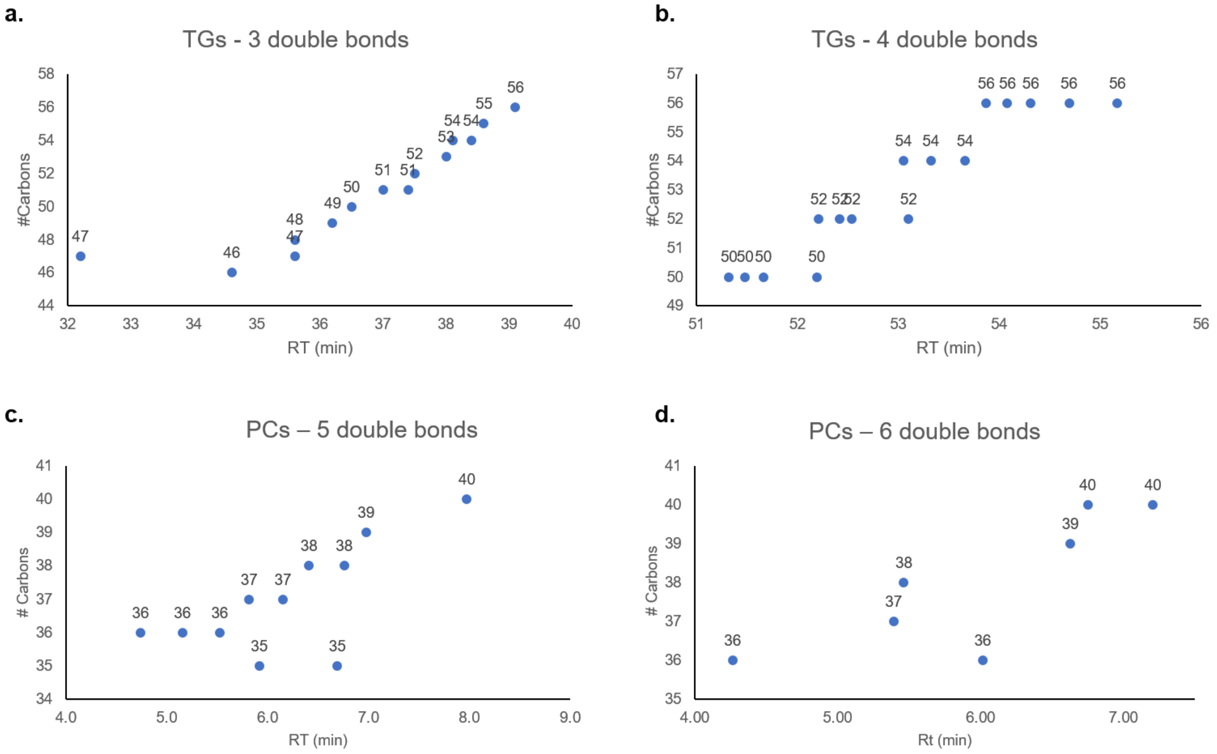

Supplementary Figure 1. Retention time behavior of lipid species as reported in the literature. a. Triacylglycerols with three double bonds[1], b. Triacylglycerols with four double bonds[2] c. Phosphatidylcholines with five double bonds[3] and d. Phosphatidylcholines with six double bonds[3],

Supplementary Figure 2

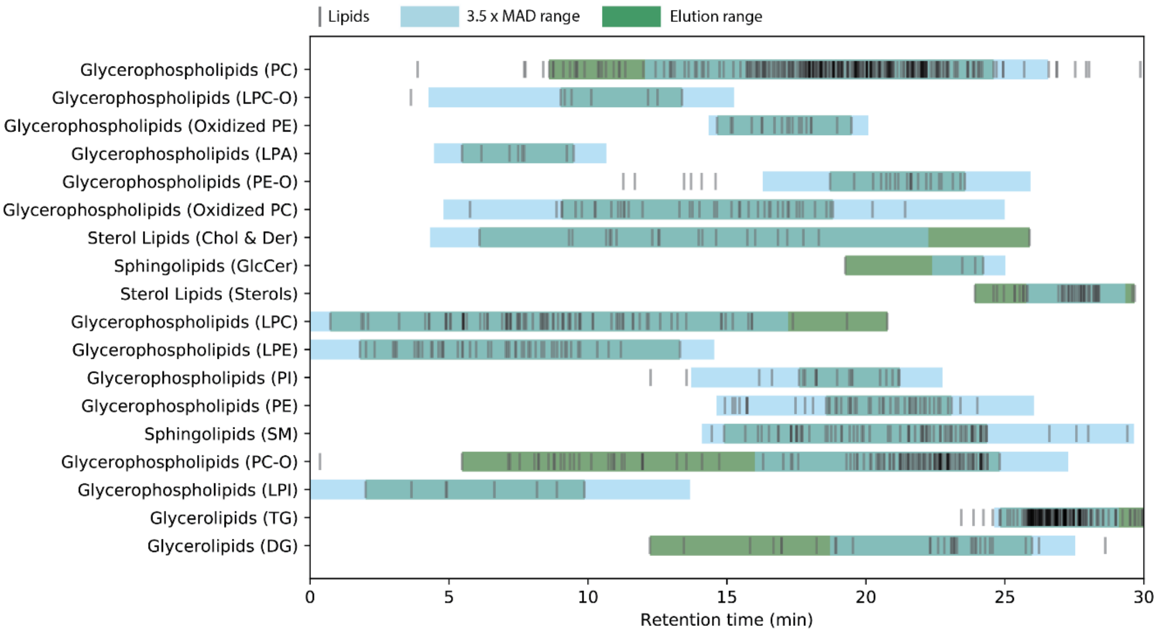

Supplementary Figure 2. Two retention time models applied to lipids detected in plasma samples. Vertical grey lines show retention times of all reported lipid species per lipid category (class). Skyblue bars indicate the retention time window corresponding to 3.5x the Median Absolute Deviation (MAD) of all lipids per lipid subclass (model 1, similar to for example ref. 4). Green bars indicate the retention time range of each lipid subclass (model 2, similar to for example ref. 5). We calculated the limits based on lipids that are in accordance with the ECN model. Opacity of the bars was set <1 to visualize overlap of the two models.

Supplementary Figure 3

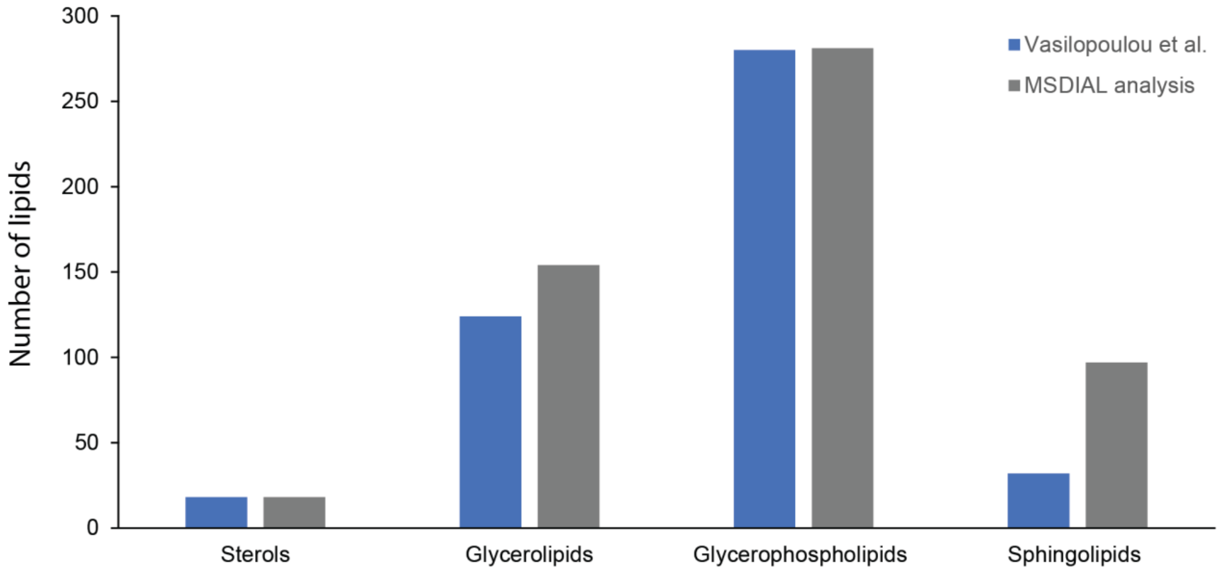

Supplementary Figure 3. Re-analysis of the human plasma raw data from Vasilopoulou et al. with MS-DIAL. Blue bars show the number of unique lipid species annotated with SimLipid as presented in the original Vasilopoulou et al. publication. Grey bars show the number of unique lipid species annotated with MS-DIAL (Supplementary Data 1). The analysis is based on the unique lipid short names as annotated from the respective software package, considering only lipid classes identified with both tools. Data from positive and negative ionization modes are merged.

Supplementary Note 1. Response to comments on specific lipid annotations (see Supplementary Data 1 in the Matters Arising article).

a. PG 17:1@_22:6. The elemental composition (raw result output by the software) corresponds to the reported mass (*m/z* 881.519) within the specified mass tolerance, and our annotation considered the FA 22:6. Please see main text for discussion of acetate adducts.
b. LPE O-16:0_0:0. This entry links to LMGP02070001, which matches the reported elemental formula and observed mass with the abbreviation LPE O-16:1 and the common name PE(P-16:0_0:0).
c. PC 15:0@_20:4. This entry matches the reported elemental composition and observed mass.
d. Oxidized PE 18:0@_20:4. This entry links to LMGP20020007, which is a lipid of the subclass oxidized glycerophosphoethanolamine and listed in LIPID MAPS with the name PE(P-18:0/20:4 (OH)). This annotation matches the reported elemental composition and observed mass (same as PE 38:4).
e. Oxidized PC 38:4. This annotation does not match the elemental composition and should read "Oxidized PC 38:5" which matches the reported elemental formula and observed mass.
f. PA 17:0_22:0@. This is a potential false positive linked to a chemically implausible fragment ion contained in the spectral library (see main text).
g. PC 15:0_16:0 (observed in negative mode) and PI & PS species. Our reporting is concordant with the procedure described in the Methods section of our original work. Please see main text for discussion.
h. Sterol lipid species (5alpha,6beta-dihydroxycholestanol, 24,25-Epoxycholesterol, Dormatinol, 24-northornasterol A). Note that we simply referred to "cholesterol and derivatives" in the main text, as this compound class typically yields non-informative fragment ions (see also LIPID MAPS reference spectra). All detected peaks are indicated in the Supplementary Table and we mentioned in the Methods section that no information about stereochemistry can be derived. The structure proposed by the commenters is not supported by further evidence.
i. PI 18:1_19:1. This diacyl phosphatidylinositol annotation is a potential false positive linked to a chemically implausible ion formation. Please see main text for further discussion.

Supplementary References

1. Triebl, A., Trötzmüller, M., Hartler, J., Stojakovic, T. & Köfeler, H. C. Lipidomics by ultrahigh performance liquid chromatography-high resolution mass spectrometry and its application to complex biological samples. *J. Chromatogr. B Anal. Technol. Biomed. Life Sci.* **1053**, 72–80 (2017).
2. Meckelmann, S. W. *et al.* Metabolic Dysregulation of the Lysophospholipid/Autotaxin Axis in the Chromosome 9p21 Gene SNP rs10757274. *Circ. Genomic Precis. Med.* **13**, (2020).
3. Koelmel, J. P. *et al.* Expanding lipidome coverage using LC-MS/MS data-dependent acquisition with automated exclusion list generation. *J Am Soc Mass Spectrom.* **28**, 908–917 (2018).
4. Hutchins, P. D., Russell, J. D. & Coon, J. J. LipiDex: An Integrated Software Package for High-Confidence Lipid Identification. *Cell Syst.* **6**, 621-625.e5 (2018).
5. Contrepois, K. *et al.* Cross-Platform Comparison of Untargeted and Targeted Lipidomics Approaches on Aging Mouse Plasma. *Sci. Rep.* **8**, 17747 (2018).
