# Supplementary material for: Reply to “Quality control requirements for the correct annotation of lipidomics data”
Source: Nat Commun. 2021 Aug 6;12:4772. doi: 10.1038/s41467-021-24985-x (PMC8346504; doi:10.1038/s41467-021-24985-x)
Supplement: Supplementary file 2 — Description of Additional Supplementary Files [file 41467_2021_24985_MOESM2_ESM.pdf]

Description of Additional Supplementary Files

File Name: Supplementary Data 1

Description: Re-analysis of the human plasma raw files from Vasilopoulou et al. with MS-DIAL v4.24. Raw files were processed using the included parameters for positive and negative ionization mode, and the result list was further curated for outliers in retention time or collisional cross section before collapsing to unique short names for further analysis.
